# Results of quality rights on human rights engagement, stigma and attitudes towards mental health among Colombian medical students

**DOI:** 10.1371/journal.pone.0319431

**Published:** 2025-02-26

**Authors:** Felipe Agudelo-Hernández, Helena Vélez-Botero, Marcela Guapacha-Montoya

**Affiliations:** 1 Facultad de Ciencias para la Salud, Universidad de Manizales, Manizales, Caldas, Colombia; 2 Departamento de Psicología, Facultad de Ciencias Humanas, Universidad Nacional de Colombia, Bogotá, Colombia; 3 Facultad de Ciencias para la Salud, Programa de Pediatría, Universidad de Caldas, Manizales, Caldas, Colombia; University of Malaga: Universidad de Malaga, SPAIN

## Abstract

**Background:**

Training healthcare professionals in human rights approaches is fundamental for humanizing medical practice and promoting patient autonomy.

**Aims:**

To evaluate the impact of Quality Rights strategy training on human rights engagement, stigma reduction and attitudes towards mental health among medical students in Colombian.

**Method:**

A pre-experimental study with pre-post measures was conducted, involving 194 medical students, during the first semester of 2024 in Manizales, Caldas, Colombia. To assess notions and commitment to human rights were used the Human Rights Exposure in Social Work and Human Rights Engagement in Social Work. Attitudes toward people with mental disorders were measured using the Community Attitudes Towards the Mentally III (CAMI) scale, and attitudes toward mental health education were assessed using the Mental Illness Clinicians’ Attitude Scale (MICA). The intervention was based on QualityRights, an initiative of the World Health Organization, which aims to improve the quality of care in mental health services and to promote the human rights of people with psychosocial disabilities. Initial data comparisons were made using the Mann-Whitney U test and the Kruskal-Wallis test. Pretest and postest data were compared using the Wilcoxon test.

**Results:**

Statistically significant improvements were observed in human rights understanding, reduced stigmatizing attitudes toward mental health and decreased authoritarianism. While students demonstrated enhanced human rights knowledge and less stigmatizing attitudes, we observed a concurrent decrease in benevolence scores.

**Conclusion:**

The Quality Rights training strategy shows promise in improving medical students’ understanding of mental health conditions and promoting empathetic practices. However, ongoing sustained and monitoring strategies are necessary to ensure long-term adoption of human rights-based attitudes and practices in healthcare settings.

## Introduction

The Universal Declaration of Human Rights, established in1948, recognizes that all human beings are born free and equal in dignity and rights. Key principles -including life, liberty, and security- ensure the enjoyment of a good life, encompassing physical and mental health [1]. Health professionals play a crucial role in promoting these rights [1]. The United Nations Convention on the Rights of Persons with Disabilities emphasizes the increased risk of fundamental rights violations among individuals with mental disorders and underscores the need for equality and inclusion in healthcare services [2].

People with mental health problems often face limited access to quality services, coercive practices, and, in extreme cases, abuse within healthcare settings meant to protect them [3,4]. The lack of research on non-coercive interventions and supports systems perpetuates these challenges. Both quantitative and qualitative research investments are essential to drive meaningful change [5].

This study examines the relationship between mental health and human rights principles, specifically investigating their combined impact on improving medical education and practice [6]. A human rights approach, serving as both a principle and tool for healthcare quality, promotes more humane care and a holistic understanding of individuals, including their contexts and coping mechanisms. This framework also empowers communities to actively participate in health-related decisions, promoting autonomy and fairness in healthcare delivery [7,8].Human rights frameworks enable individual participation in health-related decisions [7], fostering autonomy and community empowerment in healthcare delivery [9]. To achieve fair and effective healthcare, human rights principles must be integrated into both education [10] and care processes [11]. Human rights education encompasses activities that promote universal respect for fundamental freedoms [5,12] developing knowledge, skills, and understanding while fostering attitudes that contribute to a universal culture of human rights [11].

Worldwide, individuals with lived experience, families, mental health professionals, and policymakers are collaborating to improve quality, promote human rights, and reduce coercion in mental health services [5]. Although concepts of inequity, social contexts, and social determinants of health are increasingly incorporated into public health curricula [13,14], the impact of human rights education in health professions remains understudied. This knowledge gap highlights the need to examine human rights implementation in health education and practice, particularly regarding patient relationships and living conditions [15]. Human rights education aims to cultivate respect for diversity and interest in the culture and living conditions of other people [16]. Through this educational approach, healthcare professionals can enhance medical practice globally, focusing on ethical, fair, and effective care [5,6,8,14].

Following the Convention on the Rights of Persons with Disabilities [2], the World Health Organization proposed the QualityRights strategy in 2012 [3,7]. This initiative aims to improve service quality preserving the dignity of people with mental, neurological, or substance use disorders [5,7]. The toolkit comprises five core training modules covering human rights, mental health, disability, capacity, recovery, and the right to freedom from coercion, along with three specialized training modules on recovery practices, eliminating seclusion and restraint, and supporting decision-making [5,7]. Key elements include respecting individual uniqueness, promoting autonomy, advocating for rights, maintaining dignity, and fostering collaborative relationships [5]. Quality improvement and respect for human rights are fundamental to enhancing recovery strategies in healthcare settings [14]. While the QualityRights Initiative is implemented globally, few studies have examined its effectiveness in clinical or educational settings [5,17].

## Present study

International human rights standards serve as catalysts for transforming mental health care, a field historically affected by discrimination and violations of human dignity [18]. Despite the growing recognition of human rights’ importance of in health professions, Latin American contexts lack validated mechanisms to evaluate the integration of these principles in educational and clinical practice [14]. This absence of validated evaluation tools particularly impacts the assessment of medical students’ understanding and application of human rights principles in clinical settings.In Colombia, this challenge is especially significant during the country’s transformation of mental health care delivery. While Law 1616 of 2013 represents a crucial advancement in mental health legislation by mandating rights-based approaches, substantial implementation barriers persist in both clinical settings and medical education [18]. The Colombian healthcare system confronts distinct challenges in mental health service delivery, including resources scarcity in rural areas, persistent stigma within the medical community, and insufficient rights-based training for healthcare professionals [18].

To address these contextual challenges, this study evaluates the impact of Quality Rights strategy training on human rights engagement, stigma reduction, and attitudes towards mental health among Colombian medical students. We hypothesize that this educational intervention will enhance students’ comprehension of human rights principles, decrease stigmatizing attitudes, and improve their approach to mental health care delivery. This research aims to strengthen and evaluate academic initiatives that promote human rights through healthcare services. By examining Colombia’s medical education system, this research seeks to enhance the incorporation of human rights principles into healthcare training while addressing the specific challenges within the Colombian mental health care context.

## Method

### Study design

A pre-experimental study with a single group and pre-post measures was conducted was conducted from May 2, 2024, to August 30, 2024. Participants completed a virtual World Health Organization (WHO) training on the rights of persons with disabilities, complemented by in-person sensitization exercises.

This design was chosen over randomized controlled trials due to practical and ethical considerations. First, since human rights training is fundamental to medical education, withholding this training from a control group raised ethical concerns. Second, the close interaction among medical student cohorts made randomization impractical within the same academic environment, as cross-contamination between groups would be difficult to prevent. This design also enabled intensive implementation and detailed observation of changes in participant’s ‘ attitudes and knowledge.

### Participants

The study was conducted at a university in Manizales, Colombia, with 194 medical students enrolled in their final five semesters. Participants were selected through convenience sampling based on the research team’s access to the university population.

The sample comprised 54.12% female and 45.87% male students. The participants’ distribution across academic semesters was as follows: Eighth semester 28.86%, Ninth semester 4.63%, Tenth semester 23.71%, Eleventh semester 12.88% and Final semester 29.89%.

Participation was voluntary, with no academic or other incentives offered. All eligible students agreed to participate. Inclusion criteria required participants to be medical student in clinical practice, complete both pret-test and post-test scales, participate in the Quality Rights training, and provide informed consent. Students who had previously completed the training were excluded.

### Instruments

#### Human Rights Exposure in Social Work (HRXSW)/Human Rights Engagement in Social Work (HRESW).

The HRXSW scale measures students’ knowledge of and exposure to human rights principles, while the Human Rights Engagement in Social Work (HRESW) scale assesses their understanding of professional practice within a human rights framework [19]. These instruments have been validated internationally [14].

The Spanish-validated version includes: HRXSW with 16 items measuring familiarity with human rights principles, and HRESW with 25 items evaluating three domains: support for human rights principles, perceived professional relevance, and practical application [19,20]. Both scales use a seven-point Likert scale (1 = strongly disagree to 7 = strongly agree). The Spanish validation reported strong reliability with Cronbach’s alpha values of.803 (HRXSW) and.856 (HRESW) [21].

#### Community Attitudes Towards the Mentally Ill (CAMI).

The CAMI measures attitudes toward people with mental illnesses and community responses to mental health services. This instrument has been validated in Latin America [22] and Colombia [23]. The scale evaluates four domains: Authoritarianism (Views regarding coercive control), Benevolence (Sympathetic but potentially paternalistic attitudes), Social restrictiveness (Views on limiting social participation) and Mental health ideology (General perspectives on mental health).

Each domain contains ten statements (five positive, five negative), rated on a five-point Likert scale. The initial scale, internal consistency reports by subscale showed a Cronbach’s alpha of.88 for mental health ideology,.80 for social restrictiveness,.76 for benevolence, and.68 for authoritarianism [24]. The Colombian validation demonstrated reliability coefficients ranging from.59 to.80 [23].

#### Mental Illness Clinicians’ Attitude Scale (MICA).

The MICA scale is a 16-item instrument measuring health professionals’ attitudes toward mental illness [25]. Items are rated on a six-point Likert scale, with higher scores (range: 16-96) indicating more stigmatizing attitudes. The scale shows good internal consistency (α = .79) and has demonstrated sensitivity to anti-stigma interventions [26]. Latin American adaptations have maintained comparable psychometric properties [27].

### Intervention

Students completed the virtual QualityRights training under the guidance of mental health tutors. This WHO strategy [5,7] focuses on protecting the human rights of people with mental disorders and epilepsy while addressing stigma and improving healthcare quality. The initiative works to reduce discrimination, deepen understanding of human rights, and strengthen mental health awareness [7]. It promotes the development of community-based services grounded in individuals rights, avoiding coercive practices for people with mental disorders. Additionally, the strategy emphasizes civil society participation, particularly from individuals with lived experience who can contribute valuable insights to mental health decision-making and action [7]. The training is freely accessible to the public through a virtual, self-directed, asynchronous platform available in multiple languages.

### Procedure

The intervention combined virtual self-training with facilitated discussions to contextualize learning after each module. For the virtual component, participants demonstrated completion by submitting the course certificate generated upon successful completion (https://www.paho.org/es/entrenamiento-virtual-instrumento-calidad-derechos-qualityrights-oms).

The training covered several essential modules. *The Human Rights* module introduced fundamental rights established in the Universal Declaration of Human Rights while addressing stigma and discrimination. The *Human Rights, Mental Health and Disability* module explored disability concepts and the United Nations Convention on the Rights of Persons with Disabilities [2] as a framework for promoting rights. The *Legal Capacity and Right to Decide* module emphasized recovery planning and advance directives to prevent forced hospitalization, with practical exercises in developing advance care plans for psychiatric patients.

The *Ending Coercion, Violence and Abuse* module outlined strategies to eliminate coercive practices in health services and explained regional and national reporting mechanisms. The *Quality Services and Community Inclusion* module connected with Colombia’s community-based mental health rehabilitation strategy and mhGAP community components [28]. The final module, *Mental Health, Well-being and Recovery*, examined health rights within the United Nations Sustainable Development Goals framework while contextualizing Colombian mental health legislation, particularly the Mental Health Law and Statutory Health Law [29], along with mechanisms for rights enforcement.

Students participated in weekly three-hour reinforcement sessions for six weeks alongside their psychiatric clinical training. A psychiatrist with social sciences expertise led these sessions, accompanied by a faculty member, with guest appearances by a social worker and an individual with lived experience. The final study sample comprised 194 students who completed certification. The medical school subsequently incorporated this training into their tenth-semester psychiatry curriculum, though funding constraints prevented continuation of the expert guest speaker program.

### Analysis

Statistical analysis using SPSS version 26 began with normality testing through the Kolmogorov-Smirnov test. Given the non-normal distribution of data, researchers employed the Mann-Whitney U test for gender comparisons and the Kruskal-Wallis test for academic semester comparisons. The Wilcoxon test evaluated pre-post intervention differences, identifying significant changes (p < 0.05) in human rights knowledge and commitment, mental health attitudes (MICA), authoritarianism, benevolence, social restrictiveness, mental health ideology, and perceived importance of mental health.

### Ethics approval statement and patient consent statement

The study adhered to the recommendations for biomedical research outlined in the Declaration of Helsinki by the World Medical Association. It received approval from the Ethics Committee of University of Manizales, documented in minutes CBE 04 of 2024. Participants voluntarily provided informed consent written to participate in the research. Data were analyzed anonymously.

## Results

The baseline suggests that knowledge and attitudes regarding Human Rights (HR) and mental health are quite similar between men and women; the only significant difference is found in the dimension of Human Rights notions, where men show a higher score (see Table 1).

**Table 1:** Comparison of study variables by gender in the pretest.

Variable	Gender	N	Mean Rank	Sum of Rank	Mean	Mann-Whitney U	Wilcoxon W	Z	Sig.
Human Rights Notions	Female	105	82,96	8710,50	3,97	3145,500	8710,500	−3,919	<0,001
	Male	89	114,66	10204,50	4,77				
	Total	194			4,34				
Human Rights Commitment	Female	105	95,49	10026,00	5,59	4461,000	10026,000	−0,543	0,587
	Male	89	99,88	8889,00	5,66				
	Total	194			5,62				
MICA	Female	105	98,30	10321,50	3,55	4588,500	8593,500	−0,216	0,829
	Male	89	96,56	8593,50	3,52				
	Total	194			3,53				
Authoritarianism	Female	105	98,07	10297,50	2,99	4612,500	8617,500	−0,154	0,877
	Male	89	96,83	8617,50	3,01				
	Total	194			3,00				
Benevolence	Female	105	93,01	9766,00	2,98	4201,000	9766,000	−1,217	0,224
	Male	89	102,80	9149,00	3,07				
	Total	194			3,02				
Social Restrictiveness	Female	105	94,48	9920,50	2,83	4355,500	9920,500	−0,818	0,413
	Male	89	101,06	8994,50	2,91				
	Total	194			2,86				
Mental Health Ideology	Female	105	96,29	10110,00	3,08	4545,000	10110,000	−0,329	0,742
	Male	89	98,93	8805,00	3,14				
	Total	194			3,11				
Social sciences and communities can be strategic allies of health professions for the development of better mental health processes	Female	105	94,49	9921,50	5,23	4356,500	9921,500	−0,920	0,357
	Male	89	101,05	8993,50	5,30				
	Total	194			5,26				
People with mental health issues should receive the same level of care as those with physical health issues	Female	105	97,69	10257,00	5,43	4653,000	8658,000	−0,062	0,951
	Male	89	97,28	8658,00	5,40				
	Total	194			5,42				
What level of importance do you assign to promoting mental health in your daily life? (0 to 10)	Female	105	93,15	9780,50	8,24	4215,500	9780,500	−1,247	0,213
	Male	89	102,63	9134,50	8,69				
	Total	194			8,44				

When comparing baseline data based on the current semester, significant differences are observed in the variables of benevolence and the perception of social sciences as strategic allies; however, the trends are not linear. Although the absence of significant variations in other variables indicates a certain consistency in medical students’ knowledge and attitudes toward Human Rights (HR) and mental health throughout the program, there is a trend of scores increasing in most variables in the middle semesters (8th and 10th) and then decreasing in the final semesters (see Table 2).

**Table 2:** Comparison of study variables by current semester in the pretest.

Variable	Semester	N	Mean Rank	Mean	Kruskal-Wallis H	df	Sig
Human Rights Notions	8	56	99,05	4,40	2,069	4	0,723
	9	9	98,06	4,44			
	10	46	102,54	4,47			
	11	25	104,10	4,50			
	12	58	89,07	4,09			
	Total	194		4,34			
Human Rights Commitment	8	56	105,91	5,78	6,896	4	0,141
	9	9	114,17	5,98			
	10	46	105,15	5,85			
	11	25	91,48	5,26			
	12	58	83,32	5,40			
	Total	194		5,62			
MICA	8	56	106,39	3,64	4,799	4	0,309
	9	9	81,50	3,31			
	10	46	91,34	3,53			
	11	25	110,20	3,67			
	12	58	90,81	3,40			
	Total	194		3,53			
Authoritarianism	8	56	101,02	3,00	3,297	4	0,509
	9	9	97,56	2,93			
	10	46	96,15	3,06			
	11	25	112,02	3,21			
	12	58	88,91	2,87			
	Total	194		3,00			
Benevolence	8	56	100,95	3,00	15,504	4	0,004
	9	9	109,56	3,02			
	10	46	110,86	3,17			
	11	25	114,46	3,26			
	12	58	74,40	2,82			
	Total	194		3,02			
Social Restrictiveness	8	56	105,10	2,91	6,434	4	0,169
	9	9	88,89	2,72			
	10	46	105,33	2,95			
	11	25	103,26	3,04			
	12	58	82,81	2,69			
	Total	194		2,86			
Mental Health Ideology	8	56	99,88	3,13	2,803	4	0,591
	9	9	93,72	3,02			
	10	46	97,30	3,12			
	11	25	111,56	3,33			
	12	58	89,88	3,01			
	Total	194		3,11			
Social sciences and communities can be strategic allies of health professions for the development of better mental health processes	8	56	96,75	5,25	13,118	4	0,011
	9	9	107,44	5,56			
	10	46	117,89	5,65			
	11	25	82,44	4,84			
	12	58	87,00	5,10			
	Total	194		5,26			
People with mental health issues should receive the same level of care as those with physical health issues	8	56	96,21	5,45	8,037	4	0,090
	9	9	105,83	5,67			
	10	46	111,50	5,70			
	11	25	82,74	4,96			
	12	58	92,71	5,33			
	Total	194		5,42			
What level of importance do you assign to promoting mental health in your daily life? (0 to 10)	8	56	94,90	8,46	2,392	4	0,664
	9	9	112,17	9,00			
	10	46	105,36	8,85			
	11	25	92,00	8,08			
	12	58	93,87	8,17			
	Total	194		8,44			

Upon comparing the results after participation in the training process, favourable changes are observed, such as a statistically significant increase in students’ notions regarding the human rights of people with mental health conditions, a reduction in negative stigmatizing attitudes towards mental health (MICA), and a decrease in authoritarianism. Notably, there is also a decrease in benevolence (see Table 3).

**Table 3:** Pre-post comparison of study variables.

Comparison		N	Mean Rank	Sum of Rank	Pretest Mean	Postest Mean	Z	Sig.	Cohen’s R
Human Rights Notions (Postest) - Human Rights Notions (Pretest)	Negative Ranks	72	89,32	6431,00	4,34	4,83	−3,770	<0,001	0,271
	Positive Ranks	121	101,57	12290,00					
	Ties	1							
	Total	194							
Human Rights Commitment (Postest) - Human Rights Commitment (Pretest)	Negative Ranks	95	100,08	9508,00	5,62	5,56	−,574	0,566	0,041
	Positive Ranks	95	90,92	8637,00					
	Ties	4							
	Total	194							
MICA(Postest) - MICA(Pretest)	Negative Ranks	115	100,34	11539,00	3,53	3,32	−3,403	<0,001	0,244
	Positive Ranks	74	86,70	6416,00					
	Ties	5							
	Total	194							
Authoritarianism (Postest) – Authoritarianism (Pretest)	Negative Ranks	118	98,36	11607,00	3,00	2,79	−3,807	<0,001	0,273
	Positive Ranks	69	86,54	5971,00					
	Ties	7							
	Total	194							
Benevolence (Postest) – Benevolence (Pretest)	Negative Ranks	95	93,22	8855,50	3,02	2,86	−3,110	<0,001	0,223
	Positive Ranks	71	70,50	5005,50					
	Ties	28							
	Total	194							
Social Restrictiveness (Postest) - Social Restrictiveness (Pretest)	Negative Ranks	96	88,52	8498,00	2,86	2,76	−1,334	0,182	0,096
	Positive Ranks	78	86,24	6727,00					
	Ties	20							
	Total	194							
Mental Health Ideology (Postest) - Mental Health Ideology (Pretest)	Negative Ranks	104	94,74	9853,00	3,11	2,96	−2,748	0,006	0,197
	Positive Ranks	74	82,14	6078,00					
	Ties	16							
	Total	194							
Social sciences and communities can be strategic allies of health professions for the development of better mental health processes (Postest - Pretest)	Negative Ranks	44	54,65	2404,50	5,26	5,31	−1,074	0,283	0,077
	Positive Ranks	60	50,93	3055,50					
	Ties	90							
	Total	194							
People with mental health issues should receive the same level of care as those with physical health issues (Postest - Pretest)	Negative Ranks	60	53,98	3238,50	5,42	5,22	−1,691	0,091	0,121
	Positive Ranks	44	50,49	2221,50					
	Ties	90							
	Total	194							
What level of importance do you assign to promoting mental health in your daily life? (0 to 10) (Postest - Pretest)	Negative Ranks	61	68,44	4175,00	8,44	8,55	−,777	0,437	0,056
	Positive Ranks	73	66,71	4870,00					
	Ties	60							
	Total	194							

Following the training, no statistically significant differences (p < 0.05) were found in the studied variables based on gender or semester. However, the effect sizes attributable to the intervention in the pre-post comparison, estimated using Cohen’s R, are primarily small, with notable effects observed in Human Rights Notions, Mental Illness Clinicians’ Attitude (MICA), and the Authoritarianism and Benevolence subscales of the Community Attitudes Towards the Mentally Ill. This suggests that the intervention had a modest effect in these areas; while in others, such as Human Rights Commitment or the importance assigned to promoting mental health, the effect was minimal. This indicates that the observed changes, although statistically significant in some cases, are generally of small magnitude in practical terms.

## Discussion

Individuals with mental disorders frequently experience human rights violations, which significantly impact their recovery process. Health sciences education must develop strategies to effectively implement human rights framework. This study evaluated the impact of Quality Rights training on human rights engagement, stigma, and mental health attitudes toward among Colombian medical students. The results confirmed the initial hypothesis regarding improvements in human rights understanding, changes in authoritarianism and benevolence, and increased recognition of mental health’s importance in health sciences training. However, students’ commitment to implementing these rights in clinical settings remained unchanged.

Studies of human rights education in health professions reveal varying levels of implementation. European medical schools report coverage of human rights concepts in only 63% of their curricula [30]. Other institutions have incorporated perspectives from psychology, philosophy, economics, political science, and anthropology, improving theoretical knowledge while struggling to measure practical impacts [15], similar to our findings. In social work [20] and nursing [16], human rights education has successfully integrated into patient care by empowering healthcare workers to use human rights language to drive healthcare improvements.

Quality Rights implementation has faced challenges across various countries [17,30–36]. While many difficulties stem from limitations in developing intersectoral mental health approach that fulfill the CRPD requirements through basic needs and opportunities, some challenges arise from training limitations [28–30], as our research confirms.

Research findings parallel our results in several contexts. Carta et al. [33] documented significant improvements in knowledge and attitudes following intensive one-week training. However, the translation of human rights knowledge into mental health practice remained limited [30], a pattern observed across African countries [34], Latin America [31], and Europe [30,32,36]. An Indian study demonstrated stronger practical implementation through extended follow-up, monitoring changes for 12 months post-training [35].

Our finding of decreased benevolence scores merits careful consideration. Rather than indicating a negative outcome, this reduction might signal a positive shift away from paternalistic mental health care approaches. Traditional benevolence often manifests as overprotection that undermines patient autonomy, as documented in mental health stigma research [24,37]. The decrease suggests participants developed a rights-based perspective, viewing individuals with mental health conditions as autonomous agents rather than passive care recipients. This interpretation aligns with improved human rights understanding and reduced authoritarianism, reflecting movement toward recovery-oriented care approaches [38].

In this regard, Gill et al. [5] emphasize that revising and redesigning mental health curricula at both undergraduate and graduate levels is crucial for promoting alternatives to coercive practices, reducing stigma, and fostering community inclusion in mental health services. Traditional mental health education and training programs, which often present coercive practices as inevitable components of mental healthcare, need to be transformed to incorporate comprehensive training on human rights, disability awareness, and person-centered recovery approaches [3,12].

Regarding the commitment to implementing human rights principles in healthcare settings, research in contexts similar to our study indicates no significant differences when compared to professions with more extensive human rights curricula, such as social work [14]. This observation, also reflected in our findings, highlights the need to strengthen practical applications of human rights concepts beyond theoretical training. One limitation of our study is that it excludes students from disciplines other than medicine and does not address specific implementation variables within health services. Future research would benefit from extending the follow-up period to better evaluate how Quality Rights capacities translate into healthcare practice. We also recommend incorporating individuals with lived experience, their families, and informal caregivers in both developing and conducting training programs. Additionally, implementing qualitative approaches and examining healthcare experiences from a life-course perspective would provide valuable insights.

While we investigated various sociodemographic factors—including indigenous community membership, socioeconomic status, and personal and family medical history—these variables did not yield significant results in our analyses. Nevertheless, further investigation of these factors remains crucial, as they may influence how human rights principles are applied in mental health care settings.

Healthcare professionals must thoroughly understand and actively promote the Universal Declaration of Human Rights, which is built upon the fundamental principles of life, liberty, and security to ensure human dignity. These professionals are instrumental in advocating for and protecting these essential rights. Our study demonstrates promising outcomes using a comprehensive global strategy to achieve these objectives.

Incorporating Quality Rights into healthcare professional training could enhance practitioners’ capabilities and lead to improved outcomes for individuals with mental disorders. This approach promotes stakeholder collaboration, recognizes individual freedoms, encourages community participation in well-being creation, and enhances healthcare delivery processes. These results emphasize the importance of strengthening human rights education in university curricula, encompassing both theoretical knowledge and practical implementation strategies in healthcare settings.

Future initiatives could benefit from involving individuals with formal lived experience in similar educational context. While regulatory constraints in our study setting made this challenging, other studies have demonstrated significant positive outcomes from such involvement [39]. Additional strategies should include in-person training sessions, requiring the global strategy to enhance its dissemination methods.

In conclusion, our findings suggest that the Quality Rights strategy can effectively improve medical students’ perceptions of individuals with mental disorders, while also increasing the perceived importance of mental health education and enhancing understanding of human rights principles. Additional strategies are needed to improve training follow-up, strengthening both human rights commitment and the application of this framework in healthcare services. The WHO’s QualityRights program functions not as a standardized intervention but as a flexible framework, providing training and guidance to enhance services while considering local priorities, resources, and needs.

## Supporting information

S1 File Col outcomes QR. (XLSX)
